# Lionel Hersov, MD, FRCPsych

**DOI:** 10.1192/bjb.2018.66

**Published:** 2019-02

**Authors:** Philip Graham

Formerly Consultant Psychiatrist, Maudsley Hospital, UK; Professor, University of Massachusetts Medical School, USA


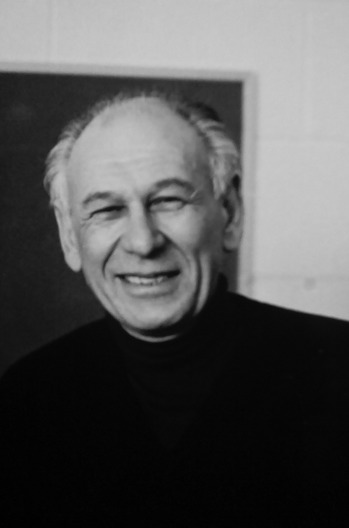


Shortly after South Africa joined the war against Germany, Lionel Hersov, who died recently at the age of 95, was 18 years old and just beginning his medical studies in Johannesburg. He volunteered for the South African Medical Corps Reserve and, from 1944 to 1945, he was attached as a medical orderly to the Royal Durban Light Infantry, 6th South African Armoured Division, and served first in the Middle East. He was then with the 5th Army Group as it fought its way up mainland Italy. His non-combatant status did not protect him against the threat of violence. On at least one occasion he was ordered to enter territory covered by enemy guns to check whether a fallen soldier had been killed or was lying wounded.

Nor was he protected from antisemitism. At another time, a German-speaking doctor pointed out to a severely wounded, captured German officer that the man treating his wounds was a Jew. The officer muttered ‘Jude, schwein’ as he spat at Lionel. He did not talk about his war experiences but, much later, visiting the battle sites in Italy with two of his children, he was deeply moved at the memory of friends who had not made it home.

At the end of the war, Lionel resumed his medical studies at the University of Witwatersrand, Johannesburg, qualifying as a doctor in 1948. Shortly after qualification, he decided to become a psychiatrist and worked in various junior posts in Johannesburg and Pretoria. However, training opportunities in psychiatry in South Africa were limited at that time. In 1952 he was accepted on the psychiatry training programme at the Maudsley Hospital, London. By 1955 he had decided to specialise in child psychiatry, and it was in this field that he made his mark. He only returned to South Africa subsequently for brief visits.

In the mid-1950s, child psychiatric practice in Britain and elsewhere was largely uninformed by scientific data. It was felt that the behaviour and emotional problems of childhood and adolescence were too complex to be studied in any systematic manner. Around that time, a small number of academically minded child psychiatrists, of whom Lionel Hersov was one of the first, decided that the time had come to apply scientific methods to the subject.

His chosen topic, which formed the basis of his MD thesis, was non-attendance at school. It was already known that children who failed to attend school fell into one of two groups. There were school refusers who were anxious about attending, either because of a fear of what might happen to them at school, such as being bullied by another student or a teacher, or because they were over-anxiously attached to their mothers.

Then there were truants who were usually rebelling against unsatisfactory home backgrounds and found little at school to interest them. Hersov was able to show that these two groups differed markedly in their relationships with their parents, their personalities, their level of academic achievement and the presence of other behaviour disorders. His findings had major implications for clinical management.^1^

After he left the Maudsley, he was appointed a consultant child psychiatrist, first at the Child Guidance Training Centre, London, and then at the Children's Hospital, Great Ormond Street, London. He returned to the Maudsley Hospital as a consultant in 1968, but also held a part-time appointment attached to the paediatric department at the Hammersmith Hospital.

While at the Maudsley, where he worked as a consultant child psychiatrist until 1994, he was an inspiring teacher and role model. Many of those who later made their mark in the field were led into it by his example. He was an astute clinician who formed excellent therapeutic relationships with the troubled children and families referred to him. While he had had experience of personal psychoanalysis, his approach was influenced not only by psychodynamic theory, but by a range of different perspectives. His colleagues found him to be a delightful man, tolerant and calm, with a talent for friendship. His warmth, humanity and wisdom were all deeply appreciated.

Shortly after the publication of papers arising from his research, he was invited to become Joint Editor of the recently founded *Journal of Child Psychology and Psychiatry and Allied Disciplines.* He acted as Senior Editor of this journal for 21 years, from 1963 to 1984. During his editorship, the journal gained so greatly in status and prestige, that, whereas in the beginning there were barely enough papers submitted to allow quarterly publication, at the time he stopped, it had an international reputation and began to appear six times a year, only accepting papers of very high quality. He retained his connection with the journal for a further 26 years, serving first as Corresponding Editor while in the United States, and then as Book Review Editor.

In 1977, he was invited by Michael Rutter to be Joint Editor of *Child Psychiatry: Modern Approaches,* the leading textbook in the field worldwide. He remained joint editor for the next two editions of this outstanding book. By now, Lionel's reputation as a leading academic had become international, and in 1978 he was elected President of the International Association of Child and Adolescent Psychiatry. The Congress held in Dublin in 1982, at the end of his presidency, was an event made memorable by the step-change in the quality of presentations.

In 1984, he moved to a position at the University of Massachusetts Medical School in Worcester, Massachusetts in the USA and remained there for over 6 years. There, he had a major influence on the teaching of child psychiatry and on clinical work. On hearing of his death, many of his former colleagues in Worcester referred to the contribution he had made there with extraordinary admiration. One wrote, ‘Lionel was a wonderful man whose support to the emerging child division was phenomenal and long-lasting’. Another noted that ‘While his expertise was child psychiatry, his knowledge went far beyond this…’ Their recollections had a lighter side. Many recalled his predilection for frozen yogurt dessert.

After his return to London in 2000, Lionel was appointed Honorary Distinguished Visiting Scientist at the Tavistock and Portman NHS Trust and took a particular interest in mentoring psychiatrists in training. Earlier, he had held a number of other significant positions. From 1976 to 1984 he was Civilian Consultant in Child and Adolescent Psychiatry to the British Army, advising on matters arising with army families. In 2011 he was awarded an Honorary Doctorate jointly by the Tavistock Clinic and the University of Essex.

Although he did not practice his religion once he reached adulthood, Lionel's parents were both Jewish. They had migrated with their families from Russia in the later years of the 19th century. His father, Charles, was a shopkeeper, and his mother, May, née Goodman, looked after the family and helped in the shop. He was the oldest of three with a younger brother and sister. He was brought up in a small Transvaal town near Pretoria, winning a place at the University of Witwatersrand, Johannesburg, to study medicine in 1940. In 1952, shortly after he came to London for postgraduate training, he married Zoe Menell, a South African graduate of Vassar College, New York. She had just completed postgraduate studies at the Sorbonne, Paris. They had four children: John, an advocate for learning disabled adults; Gregory, a theatre director; Isabelle Mary, an art gallery curator; and Martin, a media company executive.

In his younger days, Lionel was a stylish tennis player and later took up squash, which he played highly competitively. In his early 70s, however, he developed quite severe back pain which limited his mobility. He remained an enthusiastic lover of jazz. Sometime before he died, he developed Alzheimer's disease, which clouded his last years. He is survived by Zoe and his four children, as well as five grandchildren.

Lionel Hersov, child psychiatrist, was born on November 19, 1922. He died on March 11, 2018, aged 95 after a long illness.

Note: This obituary is based on one published in *The Times* on 18 May 2018.
